# Six-year post-surgical evaluation in the treatment protocols in the dental arches of children with oral cleft: longitudinal study

**DOI:** 10.1590/1678-7757-2022-0120

**Published:** 2022-07-29

**Authors:** Eloá Cristina Passucci AMBROSIO, Isabela Castro SARTORI, Paula Karine JORGE, Cleide Felício Carvalho CARRARA, Fabrício Pinelli VALARELLI, Maria Aparecida Andrade Moreira MACHADO, Thais Marchini OLIVEIRA

**Affiliations:** 1 Universidade de São Paulo Faculdade de Odontologia de Bauru Departamento de Odontopediatria, Ortodontia e Saúde Coletiva Bauru São Paulo Brasil Universidade de São Paulo, Faculdade de Odontologia de Bauru, Departamento de Odontopediatria, Ortodontia e Saúde Coletiva, Bauru, São Paulo, Brasil.; 2 Universidade de São Paulo Hospital de Reabilitação de Anomalias Craniofaciais Bauru São Paulo Brasil Universidade de São Paulo, Hospital de Reabilitação de Anomalias Craniofaciais, Bauru, São Paulo, Brasil.; 3 Universidade Ingá Departamento de Ortodonti Maringá Paraná Brasil Universidade Ingá, Departamento de Ortodontia, Maringá, Paraná, Brasil.

**Keywords:** Cleft lip, Cleft palate, Surgeons, Dental arch, Imaging, three-dimensional

## Abstract

Oral cleft surgical repairs are performed using different techniques worldwide.

**Objective:**

To evaluate and compare the development of the dental arches of children with unilateral cleft lip and palate before and after the primary surgeries performed with different techniques at the first months and six years of life.

**Methodology:**

This is a retrospective longitudinal study. The sample comprised 56 dental casts divided int the following groups: Group 1 (G1) – cheiloplasty (Millard technique) at three months and one-step palatoplasty (von Langenbeck technique) at 12 months; and Group 2 (G2) – cheiloplasty (Millard technique) and two-step palatoplasty: anterior hard palate closure (Hans Pichler technique) at three months and posterior soft palate closure (Sommerlad technique) at 12 months. The digitized dental casts were evaluated at three months – pre-surgical (T1) and six years of life– post-surgical (T2). The following linear measurements were analyzed: intercanine (C–C’), intertuberosity (T–T’) distances; anterior dental arch (I–CC’), anterior intersegment (I–C’), and total arch (I–TT’) lengths. The palate area was also measured. Parametric and non-parametric tests were applied (p<0.05).

**Results:**

In G1, the intragroup comparison showed statistically significant smaller I–CC’ and I–C’ at T2 (p=0.001 and p<0.001, respectively), while T–T’, I–TT’, and area comparisons were significantly greater (p<0.001, p=0.002, and p<0.001, respectively). In G2, the intragroup comparison exhibited statistically significant smaller C–C’ and I–C’ at T2 (p=0.004, for both), whereas T–T’, I–TT’ and area comparisons were significantly greater (p<0.001, p=0.004, and p<0.001, respectively). At T2, the intergroup analysis revealed that G1 had a statistically significant smaller I–CC’ (p=0.014). The analysis of the intergroup differences (∆=T2–T1) showed that G1 had a statistically smaller I–CC’ (p=0.043).

**Conclusion:**

The two-step palatoplasty showed a more favorable prognosis for the maxillary growth than one-step palatoplasty in children with oral clefts.

## Introduction

Cleft lip and palate (CLP) is the malformation most common diagnosed in the craniofacial region of the humans. CLP etiology is complex due to multifactorial factors such as genetic^1 , 2^ and environment^2^ , bringing an abnormal facial development during embryogenesis. This is associated to severe development anomalies of the hard and soft tissues. The maxillary growth disturbance is typical in individuals with cleft lip and palate, probably due to the lack of maxillary growth caused by the healing of the lip and/or palate repair.^3 , 4^ Children with CLP require multidisciplinary treatment since they show problems with dental anomalies, esthetics, hearing and speech impairment, and mainly, psychosocial behavior.^1 , 2^ Thousands of CLP surgical repairs are performed annually through different techniques worldwide. However, the literature lacks studies on the comparison of the outcomes of these different repair techniques. Each rehabilitation center treats CLP with different surgical approaches,^4 , 5^ with and without presurgical orthopedics^2 , 6 , 7^ , different time and techniques of primary surgeries,^2 , 7 , 8^ alveolar bone graft with different materials^9^ , and surgeon techniques and experience.^10 , 11^

In this context, cheiloplasty (lip surgical repair) is frequently performed in either newborns during the first week of life or in babies between 3 and 6 months of life. Palatoplasty (palate surgical repair), in turn, is performed between 12 and 18 months of age.^3 , 12^ The repair aims to restore the normal morphology and the function, with the minimum of disturbance of the maxillary growth potential.^7^ Studies have affirmed that the maxillary arch dimension of individuals with unilateral CLP is significantly smaller than that of the individual without oral clefts.^4^ Thus, the primary plastic surgeries rehabilitate the esthetics and function, but they caused a deleterious side-effect on the maxillary growth due to the healings from the lip/palate repair.^3^ This results in a concave face, Class III malocclusion, lack of midface growth, and orthodontics anomalies such as crowding, rotation, and tooth mispositioning.^4^ In children with unilateral CLP, cheiloplasty can be repaired by Millard technique (incisions that allowed the flat rotation and advancement) and one-step palatoplasty (hard and soft palate) by von Langenbeck technique (mucoperiosteal flaps through lateral relaxing incisions).^5^ Besides von Langenbeck technique, palate closure can be achieved by several other surgical techniques such as Hans Pichler technique (anterior palate closure alone at three months) and Sommerlad technique (posterior palate closure alone at 12 months).

Orofacial cleft rehabilitation centers work with several plastic surgeons, whose apply different surgical techniques. In a longitudinal analysis of different surgical techniques, all individuals must be operated by the same surgeon for a homogeneous result.^13^ By using tridimensional (3D) images, long-term comparisons of several surgical techniques and interventions can be obtained at different time intervals; the cleft severity can be verified by measurements to provide proper surgical planning; and dental development can be predicted.^7 , 14 , 15^

The evaluation of primary surgeries outcomes in six-year-old children allows the analysis of the dental arch dimensions and maxilla-mandible relation before the mixed dentition and the indication of other treatments, such as secondary alveolar bone graft and orthodontics.^16^ This study aims to evaluate the development of dental arches of children with unilateral cleft lip and palate. Our specific objective is to compare dental arch before and after the primary surgeries performed with different techniques at the first months and six years of life. The hypothesis proposes that different treatment protocols reach the same result in post-surgical palate development of children with oral cleft.

## Methodology

This study was approved by the Institutional Review Board under protocol 4.630.108. This retrospective longitudinal study used dental cast, which was part of the routine documentation of the center. No informed consent was necessary for parents or legal guardians. The patients were selected from the archives of the center in the period of 2010 to 2019. The inclusion criteria were children with unilateral CLP, regularly enrolled in the institution, operated by the same plastic surgeon, who started the rehabilitation treatment without previous surgery, and returned at six years-old to the hospital. Children with associated malformation or syndrome and with incomplete records were excluded.

Sample size calculation used the study of Carrara, et al.^10^ (2016), considering a standard deviation of 2.32 mm for the total dental arch length at pre-surgical stage, with 5% significance level, 80% power test, and minimum difference to be clinically detected of 2.7 mm. The minimum sample size of each group was of 14 children.

**Figure 1:**
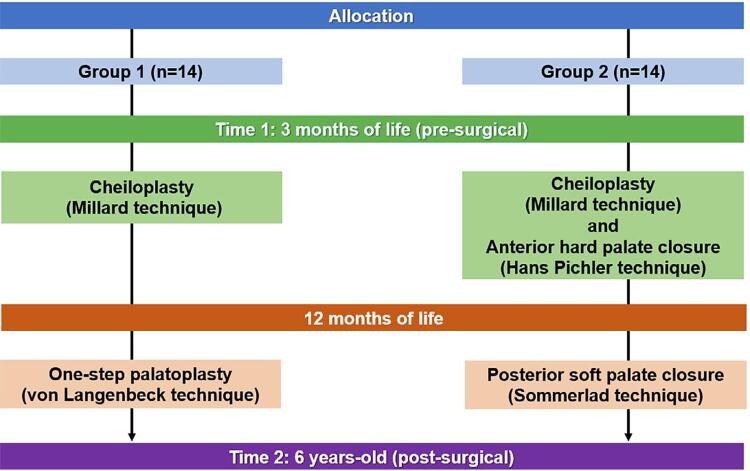
Study flowchart

The sample was divided into two groups according to the surgical technique: Group 1 (G1) – cheiloplasty (Millard technique) at 3 months and one-step palatoplasty (von Langenbeck technique) at 12 months; and Group 2 (G2) – cheiloplasty (Millard technique) and two-step palatoplasty: anterior hard palate closure (Hans Pichler technique) at 3 months and posterior soft palate closure (Sommerlad technique) at 12 months ( Figure 1 ).

The children had the impressions taken at 3 months of life (pre-surgical) – T1 and at six years-old (post-surgical) – T2. The dental casts were digitized through 3D Scanner (Scanner R700^TM^; 3Shape AS, Copenhagen, Denmark) and analyzed by two examiners by stereophotogrammetry software (Mirror imaging software, Canfield Scientific, Inc., Fairfield, NJ, USA).^10 , 12^

**Figure 2:**
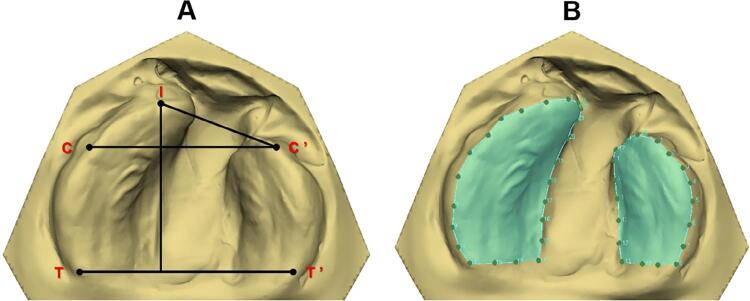
Dental arch of T1. A) Anatomical points and linear measurements. B) Contour of the greater and smaller segments to calculate the area

**Figure 3:**
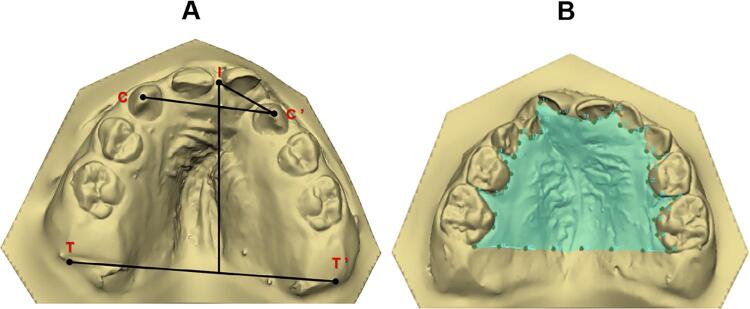
Dental arch of T2. A) Anatomical points and linear measurements. B) Contour of the palate to calculate the area

The following linear measurements were analyzed: intercanine distance (C–C’) – transversal line between the eruption points and/or cusps of the maxillary primary canine in the greater (C) and smaller bone segment (C’); anterior dental arch length (I–CC’) – straight line of the interincisive point (I) perpendicularly to the C–C’ distance; anterior intersegment length (I–C’) – straight line from the point I to the eruption point and/or cusp of the maxillary primary canine in the smaller bone segment (C’); intertuberosity distance (T–T’) – transversal line from the end of the alveolar ridge of the greater (T) to the smaller (T’) bone segments; pre-surgical sagittal length (I–TT’) – straight line from the point (I) perpendicularly to the distance T–T’.^6 , 10 , 12 , 17 , 18^ All linear measurements were analyzed in millimeters (mm) ( Figures 2A and 3A ).

The dental arch area was analyzed in squared millimeters (mm^2^). At T1, the area was marked by using the alveolar ridge crest contouring each palatal bone segment adjacent to the cleft, with posterior limit of the distance T–T’. At this time period, both segments were summed to enable the comparisons. At T2, the area was marked by contouring the primary teeth with posterior limit of the distance T–T’. ^10 , 12^ ( Figures 2B and 3B ).

All statistical analyses were performed in GraphPad Prism software (Prism 5 for Windows - Version 5.0 – GraphPad software., Inc. San Diego, USA), with a 5% level of significance. Shapiro-Wilk test was used to check the normality. To evaluate the method reliability, a third of the sample was measured twice with a 15-day interval. Wilcoxon test was used to verify the intraexaminer reliability, and Mann-Whitney test to verify the interexaminer reliability. Dahlberg’s formula quantified the casual error. Paired t-test and Wilcoxon test were used to analyze the intragroup comparisons, and the independent t-test and Mann-Whitney test to the intergroup comparisons. In the parametric analyses, data was presented as means and standard deviation (SD) and non-parametric analyses as median and interquartile amplitude (IA).

## Results

### Sample characterization

Fourteen children composed each group, totalizing 56 dental models. The mean ages were 0.33 (± 0.08) years at T1 and 6.51 (± 0.86) years at T2.

### Analyses of intraexaminer and interexaminer reliability

No statistically significant differences occurred in the analyses of the intraexaminer (Wilcoxon test, p=0.100 and Dahlberg’s formula =0.287) and interexaminer reliability (Mann-Whitney test, p=0.962).

### Intragroup analysis

In G1, the intragroup comparison showed statistically significant smaller I–CC’ and I–C’ means at T2 (p=0.001 and p<0.001, respectively) ( Table 1 ). T–T’, I–TT’, and area comparisons were significantly greater between times (p<0.001, p=0.002, and p<0.001, respectively) ( Table 1 ).

**Table 1:** Analyses of the linear measurements (mm) and area (mm2) of Group 1 – Paired t-test and Wilcoxon test (‡)

Variables	T1, Mean/ Median (SD/ IA)	T2, Mean/ Median (SD/ IA)	P-value
C – C’	29.61 (5.73)^†^	25.64 (4.15)^†^	0.104^‡^
I – CC’	6.83 (0.91)	4.31 (2.14)	0.001
I – C’	19.38 (3.13)	12.22 (2.11)	<0.001
T – T’	37.56 (4.72)^†^	46.36 (5.41)^†^	<0.001^‡^
I – TT’	25.07 (1.47)	29.2 (3.88)	0.002
Area	837.66 (161.68)^†^	1274.89 (206.15)^†^	<0.001^‡^

† Median and IA.

In G2, the intragroup comparison exhibited statistically significant smaller C–C’ and I–C’ means at T2 (p=0.004). While T–T’, I–TT’ and area comparisons were significantly greater between times (p<0.001, p=0.004, and p<0.001, respectively) ( Table 2 ).

**Table 2:** Analyses of the linear measurements (mm) and area (mm2) of Group 2 – Paired t-test and Wilcoxon test (‡)

Variables	T1, Mean/ Median (SD/ IA)	T2, Mean/ Median (SD/ IA)	P-value
C – C’	27.9 (2.95)	25.55 (2.75)	0.004^*^
I – CC’	6.76 (1.32)	6.07 (1.30)	0.252
I – C’	18.2 (3.99)	13.22 (1.02)	0.004^†*^
T – T’	36.07 (2.14)	43.33 (3.67)	<0.001^*^
I – TT’	25.4 (3.17)	29.26 (3.73)	0.004^*^
Area	860.12 (148.10)	1156.98 (158.37)	<0.001^*^

† Median and IA.

### Intergroup analyses

At T2, the intergroup analysis revealed that G1 had a statistically significant smaller I–CC’ mean than that of G2 (p=0.014) ( Table 3 ).

**Table 3:** Intergroup analysis (G1 vs. G2) of the linear measurements (mm) and area (mm2) – Independent t-test and Mann-Whitney test (§)

Variables	T1, P-value	T2, P-value
C – C’	0.800^§^	0.495
I – CC’	0.870	0.014
I – C’	0.287	0.323^§^
T – T’	0.730^§^	0.057
I – TT’	0.727	0.966
Area	0.906	0.147^§^

† Median and IA.

The analysis of the intergroup differences ( Δ = T2 – T1 ) showed that G1 had a statistically smaller I–CC’ mean than that of G2 (p=0.043) ( Table 4 ).

**Table 4:** Analyses of the intergroup differences (∆=T2–T1) of the linear measurements (mm) and area (mm2) – Independent t-test

Variables	G1, Mean (SD)	G2, Mean (SD)	P-value
C – C’	1.92 (4.01)	-2.34 (2.59)	0.741
I – CC’	2.51 (2.41)	-0.68 (2.14)	0.043
I – C’	7.16 (4.04)	-4.59 (4.61)	0.130
T – T’	10.39 (3.45)	7.26 (4.66)	0.053
I – TT’	4.13 (4.05)	3.86 (4.24)	0.866
Area	421.64 (261.33)	296.86 (207.13)	0.173

## Discussion

The treatment protocols evaluated in our study were performed by a single plastic surgeon with 35 years of experience in cheiloplasty and palatoplasty surgeries in individuals with orofacial clefts. This is very relevant criteria since the outcomes obtained are homogeneous, especially when evaluating the influence of surgical interventions. Surgeon’s experience is more important than the procedure itself in maxillary growth and development analysis.^13^

The dental arches of children with cleft lip and palate were evaluated at the first months of life and at six years of age because the literature lacks studies on evaluating the maxillary growth before the onset of the permanent dentition. Most of the longitudinal studies evaluated the maxillary changes 12-24 months after the lip and palate repair surgeries.^6 , 10 , 14 , 19 - 21^ Other studies did measure the dental arch area of children with clefts, but they did not follow the maxillary growth until five years of age.^10 , 14 , 22 - 25^ Our study revealed that children subjected to two-step palate repair had better growth than those subjected to one-step palatoplasty. The changes observed led to rejection of hypothesis since the indicated a difference in post-surgical palate development of children with oral cleft.

One-step palatoplasty (G1) showed more reduction in the anterior arch length after lip and full palate repair. This was similar to the results of the study of Haque, et al.^4^ (2020) who affirmed that in children with unilateral CLP the maxillary constriction is the main disadvantage of the standard palatoplasty procedure. Two-step palatoplasty (G2) exhibited a smaller reduction in both the anterior arch length and anterior transversal arch length.

In both groups, cheiloplasty at 3 months of age had a restrictive effect on both anterior arch length and anterior transversal arch length. This result was similar to those reported by Haque, et al.^4^ (2020), who performed different surgical techniques that inhibited the maxillary growth, especially on the anterior segment. Girinon, et al.^26^ (2019) hypothesized that cheiloplasty at six months of age would enable a better anatomic reconstruction than at three months. However, further studies are necessary to prove this hypothesis. Other studies on linear measurements revealed that the maxillary anterior area of individuals with unilateral CLP underwent transversal restriction after cheiloplasty using the decreasing of the intercanine distance, but showing and increasing of the intertuberosity distance; after palatoplasty, these distances were maintained stable.^6 , 20 , 27 , 28^

At the six-year-old post-surgical evaluation, G1 had a more restrictive effect on the anterior arch length than G2, corroborating the results of the studies of Haque and Alam^29^ (2015), and Girinon, et al.^26^ (2019), in which individuals subjected to two-step palatoplasty showed better maxillary growth, that is, one-step palatoplasty was less favorable than two-step palatoplasty. Different results showed similar maxillary deficiency in individuals subjected to lip repair compared to those subjected to lip and palate repair.^30^ Yu, et al.^31^ (2020) measured the total dental arch area of individuals with unilateral CLP before palatoplasty (at 12 months) to verify if the cleft amplitude could be considered an aggravating factor on the maxillary growth and performed cephalometric analyses of these individuals at nine years of age. Moreover, Bednar, et al.^32^ (2018) measured the total dental arch area in children with unilateral CLP and without clefts at the first months of life. These different methodologies and different measurements make the comparisons difficult.

It is difficult to obtain dental casts of newborns because of the affliction and agitation of the baby during the impression procedure.^33^ Moreover, dental cast may have defect as bubbles and poorly finishing that confuse landmark, so they were eliminated of the sample. Despite these limitations, the impression procedure of newborns is the gold standard for the documentation of children with CLP. In the future, intraoral scanning could replace the impressions, but the current scanning device tips are still too big to be used inside the babies’ mouths. In this study, the software used showed good reproducibility to determine the maxillary growth.

Our study enabled a better understanding of the effect of lip and palate surgical repair on craniofacial growth and development. Notwithstanding, further studies are necessary aiming at decreasing the iatrogenic effects of the surgeries, favoring the rehabilitation, and improving the quality of life of these children. This would provide objective estimates of the maxillary growth and the outcomes could be used as control data for studies evaluating the growth and treatment of individuals with cleft lip and palate compared to those without clefts.

## Conclusion

Based on the results, this study showed that two-step palatoplasty was a more favorable prognosis for the maxillary growth than one-step palatoplasty in children with oral clefts.

## References

[1] Grassia V, Lombardi A, Kawasaki H, Ferri C, Perillo L, Mosca L, et al. Salivary micrornas as new molecular markers in cleft lip and palate: a new frontier in molecular medicine. Oncotarget 2018;9(27):18929-38. 10.18632/oncotarget.24838 PMC5922367 29721173

[2] Freitas JA, Neves LT, Almeida AL, Garib DG, Trindade-Suedam IK, Yaedú RY, et al. Rehabilitative treatment of cleft lip and palate: experience of the Hospital for Rehabilitation of Craniofacial Anomalies/USP (HRAC/USP)--Part 1: overall aspects. J Appl Oral Sci. 2012;20(1):9-15. doi: 10.1590/s1678-7757201200010000

[3] Hoffmannova E, Moslerová V, Dupej J, Borský J, Bejdová Š, Velemínská J. Three-dimensional development of the upper dental arch in unilateral cleft lip and palate patients after early neonatal cheiloplasty. Int J Pediatr Otorhinolaryngol. 2018;109:1-6. doi: 10.1016/j.ijporl.2018.03.009 10.1016/j.ijporl.2018.03.009 29728158

[4] Haque S, Khamis MF, Alam MK, Ahmad WM. Effects of multiple factors on treatment outcome in the three-dimensional maxillary arch morphometry of children with unilateral cleft lip and palate. J Craniofac Surg. 2020;31(6):e534-8. doi: 10.1097/SCS.0000000000006464 10.1097/SCS.0000000000006464 32371703

[5] Ambrosio EC, Sforza C, Menezes M, Carrara CF, Soares S, Machado MA, et al. Prospective cohort 3D study of dental arches in children with bilateral orofacial cleft: assessment of volume and superimposition. Int J Paediatr Dent. 2021;31(5):606-12. doi: 10.1111/ipd.12731 10.1111/ipd.12731 32970887

[6] Jorge PK, Gnoinski W, Vaz Laskos K, Carrara CF, Garib DG, Ozawa TO, et al. Comparison of two treatment protocols in children with unilateral complete cleft lip and palate: tridimensional evaluation of the maxillary dental arch. J Craniomaxillofac Surg. 2016;44(9):1117-22. doi: 10.1016/j.jcms.2016.06.032 10.1016/j.jcms.2016.06.032 27460947

[7] Morioka D, Mandrano N, Fujimoto H, Koga Y, Sato N, Tosa Y, et al. Longitudinal follow-up of individuals with cleft lip using three-dimensional stereophotogrammetry. J Craniofac Surg. 2018;29(5):1261-5. doi: 10.1097/SCS.0000000000004434 10.1097/SCS.0000000000004434 29521745

[8] Nicol M, Boutray M, Captier G, Bigorre M. Primary cheilorhinoseptoplasty using the Talmant protocol in unilateral complete cleft lip: functional and aesthetic results on nasal correction and comparison with the Tennison-Malek protocol. Int J Oral Maxillofac Surg. 2022:S0901-5027(22)00143-6. doi: 10.1016/j.ijom.2022.04.004 10.1016/j.ijom.2022.04.004 35523693

[9] Scalzone A, Flores-Mir C, Carozza D, d’Apuzzo F, Grassia V, Perillo L. Secondary alveolar bone grafting using autologous versus alloplastic material in the treatment of cleft lip and palate patients: systematic review and meta-analysis. Prog Orthod. 2019;20(1):6. doi: 10.1186/s40510-018-0252-y 10.1186/s40510-018-0252-y PMC6369233 30740615

[10] Carrara CF, Ambrosio EC, Mello BZ, Jorge PK, Soares S, Machado MA, et al. Three-dimensional evaluation of surgical techniques in neonates with orofacial cleft. Ann Maxillofac Surg. 2016;6(2):246-50. doi: 10.4103/2231-0746.200350 10.4103/2231-0746.200350 PMC5343636 28299266

[11] Williams AC, Sandy JR. Risk factors for poor dental arch relationships in young children born with unilateral cleft lip and palate. Plast Reconstr Surg. 2003;111(2):586-93. doi: 10.1097/01.PRS.0000041946.98451.FB 10.1097/01.PRS.0000041946.98451.FB 12560679

[12] Ambrosio EC, Sforza C, Menezes M, Gibelli D, Codari M, Carrara CF, et al. Longitudinal morphometric analysis of dental arch of children with cleft lip and palate: 3D stereophotogrammetry study. Oral Surg Oral Med Oral Pathol Oral Radiol. 2018;126(6):463-8. doi: 10.1016/j.oooo.2018.08.012 10.1016/j.oooo.2018.08.012 30249537

[13] Chong DK, Portnof JE, Xu H, Salyer KE. Reviewing the orthognathic surgical care of the patient with cleft lip and palate: the single surgeon experience. J Craniofac Surg. 2009;20 Suppl 2:1895-904. doi: 10.1097/SCS.0b013e3181b6c69f 10.1097/SCS.0b013e3181b6c69f 19816372

[14] Menezes M, Cerón-Zapata AM, López-Palacio AM, Mapelli A, Pisoni L, Sforza C. Evaluation of a three-dimensional stereophotogrammetric method to identify and measure the palatal surface area in children with unilateral cleft lip and palate. Cleft Palate Craniofac J. 2016;53(1):16-21. doi: 10.1597/14-076 10.1597/14-076 25794014

[15] Botticelli S, Pedersen TK, Küseler A, Nørholt SE, Cattaneo PM. Novel 3-D analysis for the assessment of cleft dimensions on digital models of infants with unilateral cleft lip and palate. Cleft Palate-Craniofacial J. 2019;56(1):127-33. 10.1177/1055665618770795 29652538

[16] Rando GM, Ambrosio EC, Jorge PK, Prado DZ, Falzoni MM, Carrara CF, et al. Anthropometric analysis of the dental arches of five-year-old children with cleft lip and palate. J Craniofac Surg. 2018;29(6):1657-60. doi: 10.1097/SCS.0000000000004806 10.1097/SCS.0000000000004806 30028406

[17] Maulina I, Priede D, Linkeviciene L, Akota I. The influence of early orthodontic treatment on the growth of craniofacial complex in deciduous occlusion of unilateral cleft lip and palate patients. Stomatologija. 2007;9(3):91-6. 17993742

[18] Mello BZ, Ambrosio EC, Jorge PK, Menezes M, Carrara CF, Soares S, et al. Analysis of dental arch in children with oral cleft before and after the primary surgeries. J Craniofac Surg. 2019;30(8):2456-8. doi: 10.1097/SCS.0000000000005775 10.1097/SCS.0000000000005775 31369497

[19] Cerón-Zapata AM, López-Palacio AM, Rodriguez-Ardila MJ, Berrio-Gutiérrez LM, Menezes M, Sforza C. 3D evaluation of maxillary arches in unilateral cleft lip and palate patients treated with nasoalveolar moulding *vs* . Hotz’s plate. J Oral Rehabil. 2016;43(2):111-8. doi: 10.1111/joor.12350 10.1111/joor.12350 26404105

[20] Falzoni MM, Jorge PK, Laskos KV, Carrara CF, Machado MA, Valarelli FP, et al. Three-dimensional dental arch evaluation of children with unilateral complete cleft lip and palate. Dent Oral Craniofac Res. 2016;2(2):238-41. 10.15761/DOCR.1000154

[21] Hoffmannova E, Bejdová Š, Borský J, Dupej J, Cagáňová V, Velemínská J. Palatal growth in complete unilateral cleft lip and palate patients following neonatal cheiloplasty: classic and geometric morphometric assessment. Int J Pediatr Otorhinolaryngol. 2016;90:71-6. doi: 10.1016/j.ijporl.2016.08.028 10.1016/j.ijporl.2016.08.028 27729158

[22] Lo LJ, Wong FH, Chen YR, Lin WY, Ko EW. Palatal surface area measurement: comparisons among different cleft types. Ann Plast Surg. 2003;50(1):18-23. doi: 10.1097/00000637-200301000-00004 10.1097/00000637-200301000-00004 12545104

[23] Russell LM, Long RE Jr, Romberg E. The Effect of cleft size in infants with unilateral cleft lip and palate on mixed dentition dental arch relationship. Cleft Palate Craniofac J. 2015;52(5):605-13. doi: 10.1597/13-325 10.1597/13-325 25642966

[24] Generali C, Primozic J, Richmond S, Bizzarro M, Flores-Mir C, Ovsenik M, et al. Three-dimensional evaluation of the maxillary arch and palate in unilateral cleft lip and palate subjects using digital dental casts. Eur J Orthod. 2017;39(6):641-5. doi: 10.1093/ejo/cjx019 10.1093/ejo/cjx019 28371800

[25] Darvann TA, Hermann NV, Ersbøll BK, Kreiborg S, Berkowitz S. Palatal surface area of maxillary plaster casts--a comparison between two-dimensional and three-dimensional measurements. Cleft Palate Craniofac J. 2007;44(4):381-90. doi: 10.1597/05-213.1 10.1597/05-213.1 17608546

[26] Girinon F, Ketoff S, Hennocq Q, Kogane N, Ullman N, Kadlub N, et al. Maxillary shape after primary cleft closure and before alveolar bone graft in two different management protocols: a comparative morphometric study. J Stomatol Oral Maxillofac Surg. 2019;120(5):406-9. doi: 10.1016/j.jormas.2019.02.001 10.1016/j.jormas.2019.02.001 30763782

[27] Rousseau P, Metzger M, Frucht S, Schupp W, Hempel M, Otten JE. Effect of lip closure on early maxillary growth in patients with cleft lip and palate. JAMA Facial Plast Surg. 2013;15(5):369-73. doi: 10.1001/jamafacial.2013.335 10.1001/jamafacial.2013.335 23867920

[28] Sakoda KL, Jorge PK, Carrara CF, Machado MA, Valarelli FP, Pinzan A, et al. 3D analysis of effects of primary surgeries in cleft lip/palate children during the first two years of life. Braz Oral Res. 2017;31:e46. doi: 10.1590/1807-3107BOR-2017.vol31.0046 10.1590/1807-3107BOR-2017.vol31.0046 28591242

[29] Haque S, Alam MK. Spectrum of palatoplasty has detrimental effect on maxillary growth: myth or fact? Bangladesh J Med Sci. 2015;14(1):109-10. 10.3329/bjms.v14i1.20926

[30] Li Y, Shi B, Song QG, Zuo H, Zheng Q. Effects of lip repair on maxillary growth and facial soft tissue development in patients with a complete unilateral cleft of lip, alveolus and palate. J Craniomaxillofac Surg. 2006;34(6):355-61. doi: 10.1016/j.jcms.2006.03.005 10.1016/j.jcms.2006.03.005 16859911

[31] Yu Q, Deng Q, Fu F, Li R, Zhang W, et al. A novel splicing mutation of ARHGAP29 is associated with nonsyndromic cleft lip with or without cleft palate. J Matern Fetal Neonatal Med. 2022;35(13):2499-506. doi: 10.1080/14767058.2020.1786523 10.1080/14767058.2020.1786523 32698641

[32] Bednar KA, Briss DS, Bamashmous MS, Grayson BH, Shetye PR. Palatal and alveolar tissue deficiency in infants with complete unilateral cleft lip and palate. Cleft Palate Craniofac J. 2018;55(1):64-9. doi: 10.1177/1055665617718545 10.1177/1055665617718545 34162056

[33] Bruggink R, Baan F, Kramer GJ, Maal TJ, Kuijpers-Jagtman AM, Bergé SJ, et al. Three dimensional maxillary growth modeling in newborns. Clin Oral Investig. 2019;23(10):3705-12. doi: 10.1007/s00784-018-2791-5 10.1007/s00784-018-2791-5 30635787

